# Assessing the tropical forest cover change in northern parts of Sonitpur and Udalguri District of Assam, India

**DOI:** 10.1038/s41598-021-90595-8

**Published:** 2021-06-02

**Authors:** Ranjit Mahato, Gibji Nimasow, Oyi Dai Nimasow, Dhoni Bushi

**Affiliations:** 1 Department of Geography, Rajiv Gandhi University, Doimukh, Arunachal Pradesh India; 2 Department of Botany, Rajiv Gandhi University, Doimukh, Arunachal Pradesh India

**Keywords:** Ecology, Environmental sciences

## Abstract

Sonitpur and Udalguri district of Assam possess rich tropical forests with equally important faunal species. The Nameri National Park, Sonai-Rupai Wildlife Sanctuary, and other Reserved Forests are areas of attraction for tourists and wildlife lovers. However, these protected areas are reportedly facing the problem of encroachment and large-scale deforestation. Therefore, this study attempts to estimate the forest cover change in the area through integrating the remotely sensed data of 1990, 2000, 2010, and 2020 with the Geographic Information System. The Maximum Likelihood algorithm-based supervised classification shows acceptable agreement between the classified image and the ground truth data with an overall accuracy of about 96% and a Kappa coefficient of 0.95. The results reveal a forest cover loss of 7.47% from 1990 to 2000 and 7.11% from 2000 to 2010. However, there was a slight gain of 2.34% in forest cover from 2010 to 2020. The net change of forest to non-forest was 195.17 km^2^ in the last forty years. The forest transition map shows a declining trend of forest remained forest till 2010 and a slight increase after that. There was a considerable decline in the forest to non-forest (11.94% to 3.50%) from 2000–2010 to 2010–2020. Further, a perceptible gain was also observed in the non-forest to the forest during the last four decades. The overlay analysis of forest cover maps show an area of 460.76 km^2^ (28.89%) as forest (unchanged), 764.21 km^2^ (47.91%) as non-forest (unchanged), 282.67 km^2^ (17.72%) as deforestation and 87.50 km^2^ (5.48%) as afforestation. The study found hotspots of deforestation in the closest areas of National Park, Wildlife Sanctuary, and Reserved Forests due to encroachments for human habitation, agriculture, and timber/fuelwood extractions. Therefore, the study suggests an early declaration of these protected areas as Eco-Sensitive Zone to control the increasing trends of deforestation.

## Introduction

Tropical forests constitute less than 10% of the Earth’s surface^1^ and possess about 50% of terrestrial species^2^. These forests are currently undergoing large-scale deforestation, which threatens species survival and diminishes biodiversity^3^. The annual global deforestation rate of humid tropical forests was estimated at 0.5% from 1990 to 1997, with a high yearly deforestation rate in Southeast Asia^4^. Large-scale deforestations occurred in India during the past^5^. The annual loss of forest cover in India during the decade 1990 to 2000 was estimated at 380.89 km^2^ by the Food and Agricultural Organization (FAO)^6^. High population growth rate, expansion of agricultural lands, settlements, and human-induced changes caused widespread damage to the Indian forests^7,8^. Hence, the Ministry of Environment, Forest and Climate Change (MoEFCC), Government of India has declared a number of protected areas (National Parks, Sanctuaries, and Reserved Forests) to control deforestation and degradation of forest ecology.

Assam, one of the largest States of North East India, falls in the tropical climate belt with rich biological diversity. The forests of Assam belong to seven forest type groups and can be further divided into 25 different forest types^9^. The State also possesses a host of endangered and rare mammal, avian and amphibian species like the One-Horned Rhino, pigmy hog, hispid hare, white-winged wood duck, great Indian hornbill, and many others. The total recorded forest of the State was 26,832 km^2^ (34.21%) out of the total geographical area, and 17,864 km^2^ was Reserved Forest, while 8968 km^2^ was Unclassed Forest^10^. In terms of forest canopy density classes, the State has 2,794.86 km^2^ under very dense forest, 10,278.91 km^2^ under moderately dense forest, and 15,252.74 km^2^ under open forest. Owing to the large geographical area under forest cover and rich biodiversity, 5 National Parks and 18 Wildlife Sanctuaries were declared to protect various species of flora and fauna. However, large-scale deforestation and encroachment of forest areas are at large in the State during the recent past. According to Assam Times^11^, an area of 3,396 km^2^ of the Reserved Forests across the State is under encroachment officially, and more than four lakh people have illegally settled inside the 20 Wildlife Sanctuaries and 271 Reserved Forests. In Sonitpur district, about 892 km^2^ of the forest area was under encroachment. The Sonai-Rupai Wildlife Sanctuary (WLS) has lost over 85 km^2^ of its forest, and Nameri National Park (NP) registered a sharp decline in the dense forest due to habitat fragmentation and encroachment by small farmers^12^. Similarly, villages of the Udalguri district located along the border of Bhutan show massive deforestation through logging and expansion of small tea gardens^13^.

Remote Sensing (RS) and Geographic Information System (GIS) are effective techniques for estimating forest cover change. RS and GIS techniques are used extensively for generating valuable information on forest cover in the tropical areas, land use changes over large areas^14–19^ and protected areas^12,20–23^. So far, habitat loss in Kameng and Sonitpur Elephant Reserves^24^, large-scale deforestation in Sonitpur district^15^, and land use/land cover change and fragmentation in Nameri Tiger Reserve^12^ are available. However, the areal coverage and temporal scale of the present study are different from the earlier studies. The forest cover loss in and around the study area has been reported till 2007. Hence, in the backdrop of continuing deforestation and illegal encroachments, this study attempts to estimate the forest cover changes in the study area till 2020 and make a comparative assessment of deforestation during the past four decades (1990, 2000, 2010, and 2020).

## Materials and methods

### Study area

**Figure 1:**
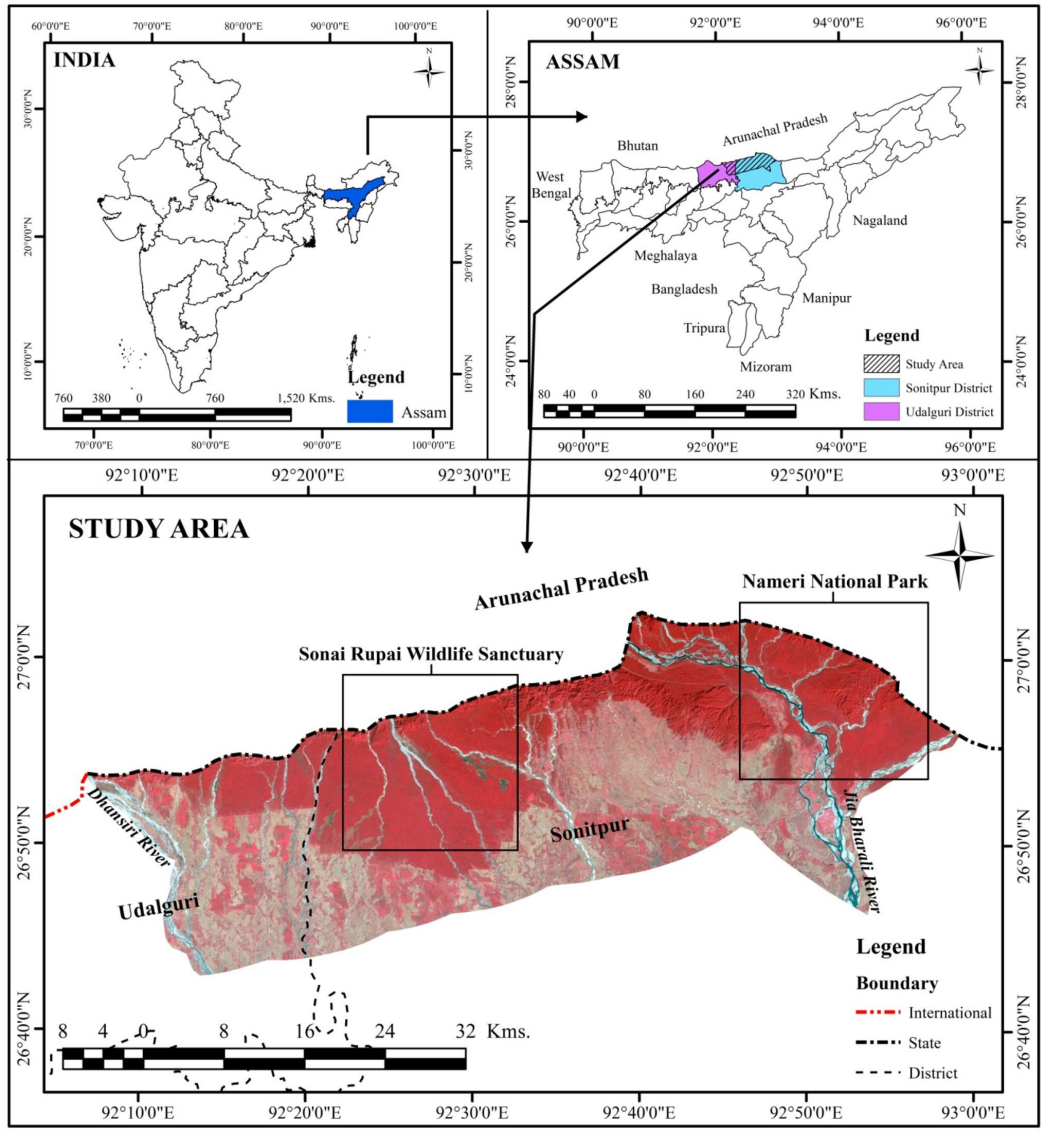
Location of the study area in Assam, India (Landsat image of 1990). Generated by the authors using ArcGIS 10.3, http://esri.com.

The study area lies in between 26° 40′ 18″ N to 27° 02′ 34″ N latitudes and 92° 06′ 48″ E to 92° 59′ 11″ E longitudes covering an area of 1939.25 km^2^ under Sonitpur and Udalguri district of Assam, India. The Area of Interest (AOI) was created by a 20 km buffer starting from the northern State boundary towards the south, and the east–west extension stretches in between *Jia Bharali* in the east to *Dhansiri* River in the west (Fig. 1). Sonitpur district is bounded in the north by the hills of Arunachal Pradesh, River Brahmaputra in the south, Bishwanath district in the east, and Udalguri and Darrang district in the west. The district covers an area of 2076.70 km^2^ with a total population of 10,19,406^25^. Udalguri district, (a Bodoland Territorial Autonomous Districts, Assam) is bounded in the north by the hills of Bhutan and Arunachal Pradesh, Darrang district in the south, Sonitpur district in the east, and Baksa district in the west. The total geographical area is above 1985.68 km^2^, with a population of 8,31,668^25^. The Nameri NP, Sonai-Rupai WLS, and other Reserved Forest areas of Assam lie in the study area. The majority of the inhabitants belong to the Bodo tribe, and other ethnic groups comprise Mishing, Garo, Karbi, Nepali, Adivasi, and non-tribal populations^26,27^.

### Database

Landsat 5—Thematic Mapper, Landsat 7—Enhanced Thematic Mapper Plus and Landsat 8—Operational Land Imager satellite data of 1990, 2000, 2010, and 2020 were used in this study to estimate the forest cover changes (Table 1). Landsat images are available free of cost from the United States Geological Survey browser Earth Explorer (http://earthexplorer.usgs.gov) and have an adequate resolution (spectral and spatial) to study forest cover changes^28^. The images were chosen from January and February with a difference of 22 days only to maintain homogeneity.

**Table 1:** Details of landsat images used in the study.

Year	Data type	Spatial resolution	Path/Row	Acquisition Date
1990	Landsat 5—TM	30 meter	136/041 & 136/042	16th January 1990
2000	Landsat 5—TM			12th January 2000
2010	Landsat 7—ETM +			31st January 2010
2020	Landsat 8—OLI			4th February 2020

### Pre-processing, classification and forest cover mapping

The downloaded Landsat images were clipped to a 20 km buffer AOI using ArcGIS 10.3. All Landsat 7 scenes collected since 30th May 2003 have 20–25% data gaps due to the Scan Line Corrector (SLC) failure^29^. Hence, the data gap of the Landsat 7—ETM + image of 2010 was corrected using Landsat Toolbox operation ‘Fix Landsat 7 Scan Line Errors’ in ArcGIS. The orthorectified images were classified by following the maximum likelihood algorithm of the supervised classification technique in ERDAS Imagine version 2014 because it is used most often for the quantitative analysis and does not require extensive training process^30,31^. The classified raster images were converted to polygons, and the misclassified pixels were corrected using cut polygon and field calculator tools. Accuracy assessment of a thematic map is essential to quantify the quality of data for intended applications by the map users^32^. The overall accuracy, producer’s accuracy, user’s accuracy, and Kappa coefficient were derived from the error matrix. The overall accuracy was computed by dividing the total correct pixels (the sum of the major diagonal) by the total number of reference pixels. The producer’s accuracy relates to the probability of a reference pixel correctly being classified (omission error). In contrast, the user’s accuracy is indicative of the likelihood that a sample pixel classified on the map represents that category on the ground actually or commission error^33^. Kappa coefficient expresses the proportionate reduction in error generated by a classification process compared to the error of a completely random classification^34^. Kappa coefficient is computed by the following Eq.^35^:

$$\widehat{k}= \frac{N\sum_{i=1}^{r}{x}_{ii}- \sum_{i=1}^{r}\left({x}_{i+}\bullet {x}_{+i}\right)}{{N}^{2}- \sum_{i=1}^{r}({x}_{i+ }\bullet {x}_{+i})}$$

where, *r* = number of rows, columns in the error matrix, *N* = total number of observations in the error matrix, *x*_*ii*_ = major diagonal element for class *i, x*_*i*+_  = total number of observations in row *i* (right margin), *x*_+*i*_ = total number of observations in column *i* (bottom margin).

**Figure 2:**
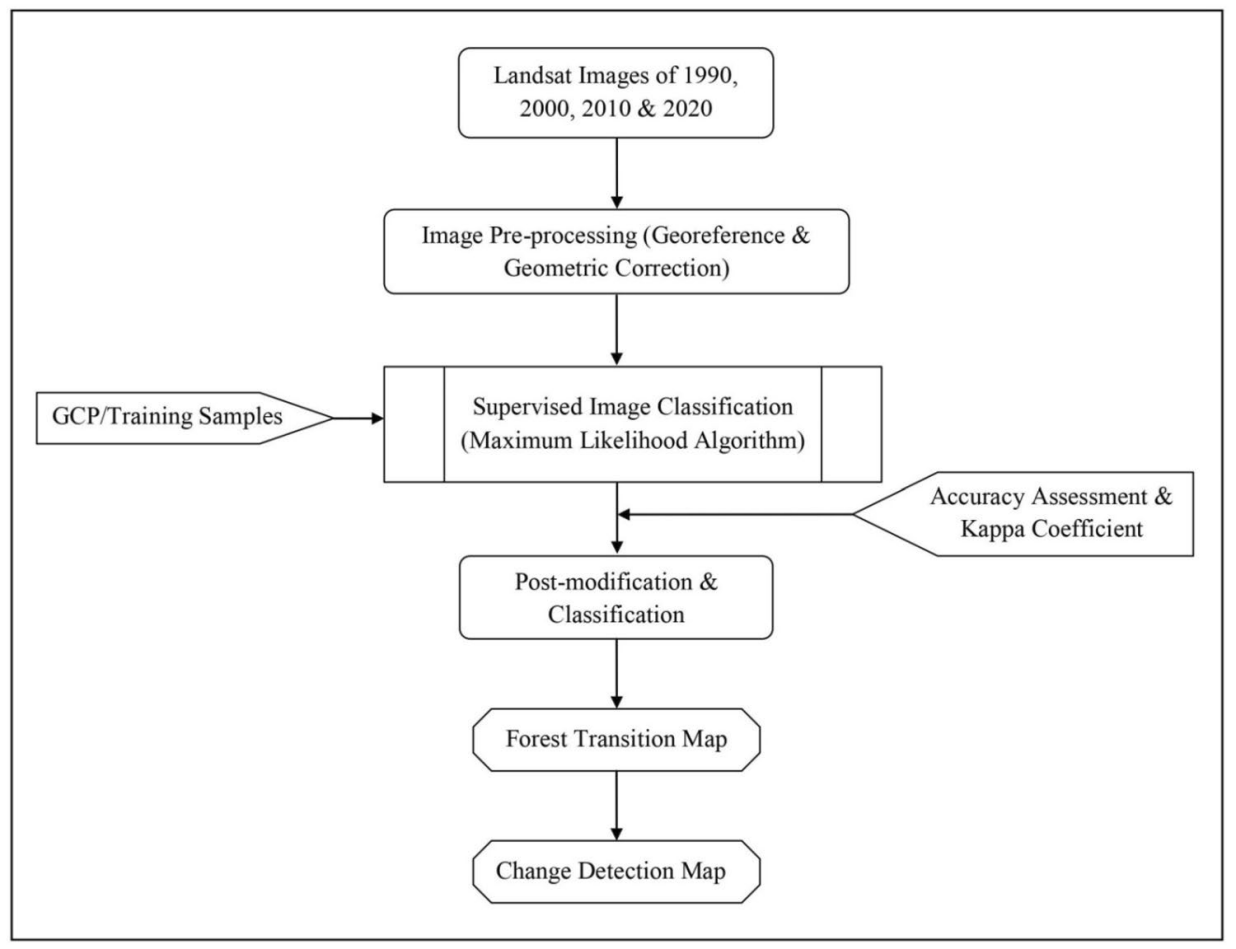
Methodology followed in the study.

As per Kappa interpretation guidelines of Landis and Koch^36^, the coefficient values range from + 1 (perfect agreement) to − 1 (complete disagreement). Finally, the thematic maps (forest cover, transition in forest cover, and net forest cover change) were derived using analysis tools (overlay/intersect) in ArcGIS (Fig. 2).

## Results

### Forest cover in the study area

**Figure 3:**
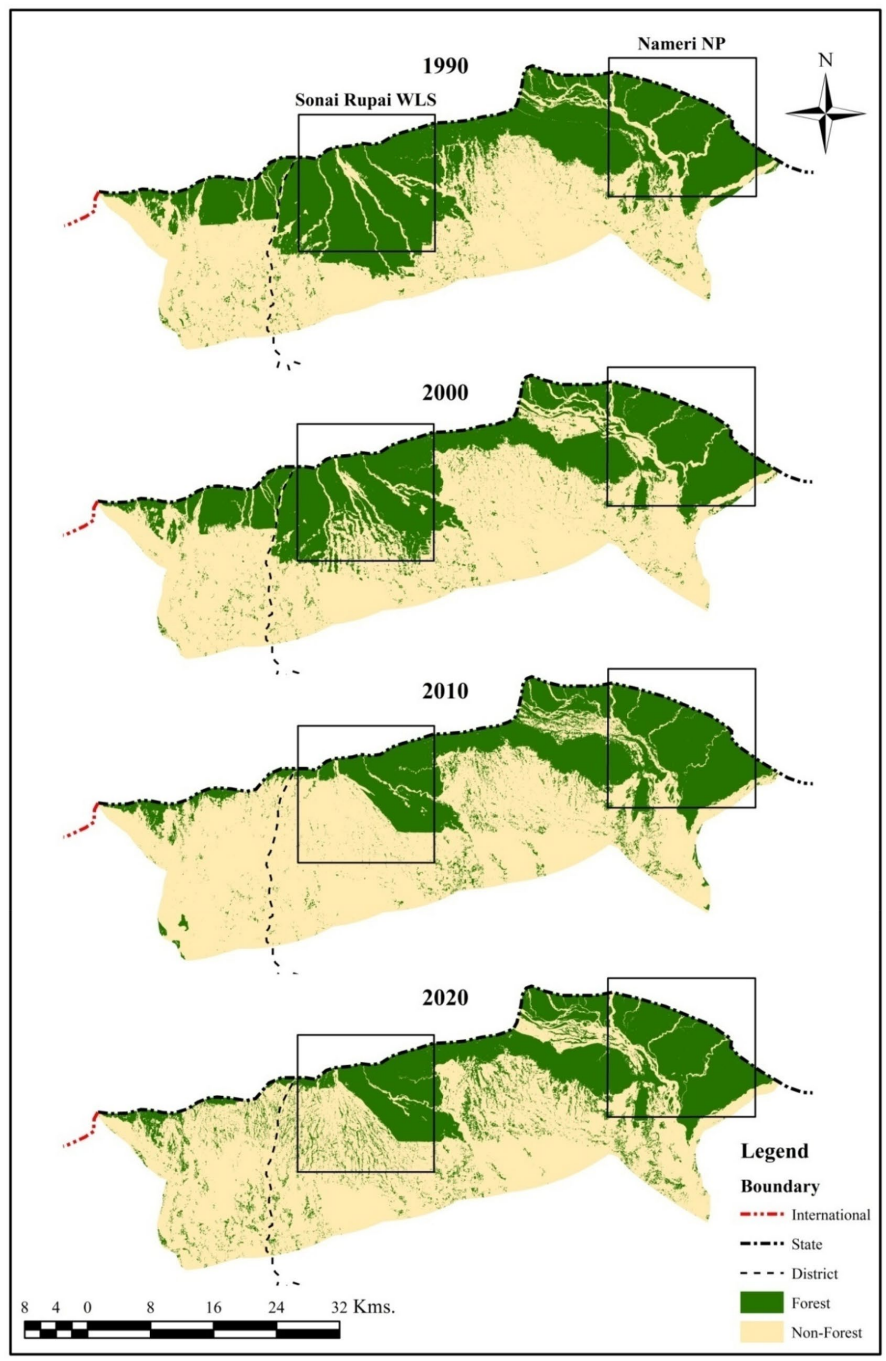
Forest cover map of 1990, 2000, 2010 and 2020 (Generated by the authors using ERDAS Imagine version 2014, http://hexagongeospatial.com).

The generated forest cover maps for 1990, 2000, 2010, and 2020 show the Forest and Non-Forest categories (Table 2 & Fig. 3). Out of the total geographical area of 1595.14 km^2^, the Forest area was 743.42 km^2^ (46.61%), and Non-Forest was 851.72 km^2^ (53.39%) in the year 1990. The forest cover declined to 624.34 km^2^ during 2000 and further reduced to 510.96 km^2^ in 2010 (Fig. 3). The forest cover loss was 7.47% from 1990 to 2000 and 7.11% from 2000 to 2010. However, the forest cover slightly increased to 548.25 km^2^ from 2010 to 2020, with a gain of 2.34% compared to the last decade (Fig. 3). Overall, the net change in forest cover was 195.17 km^2^ over the forty years (Table 2).

**Table 2:** Area under forest/non-forest and net change from 1990 to 2020.

Forest cover	1990	2000	2010	2020	Net change (km^2^)
	Area (km^2^)	Area (km^2^)	Area (km^2^)	Area (km^2^)	
Forest	743.42	624.34	510.96	548.25	(–) 195.17
Non-forest	851.72	970.80	1084.18	1046.89	( +) 195.17
**Total**	**1595.14**	**1595.14**	**1595.14**	**1595.14**	**–**

### Accuracy assessment of forest cover map

Accuracy assessment of a classification scheme is essential to validate the information derived from remotely sensed and ground truth data. Accuracy assessment was performed using 400 random points collected from the downloaded satellite images and high-resolution images of Google Earth for the representative years (1990, 2000, 2010, and 2020). The generated random points served as ground truth data for the study, which was compared with the classified images and statistically analyzed using error matrices. The accuracy assessments of the four classified images are given in Table 3. The producer’s accuracy range between 95 to 98.02%, while the user’s accuracy range between 93.75 to 97.97%. The overall accuracy was in the order of 1990 (97.50%), 2020 (97.25%), 2010 (95.50%), and 2000 (95.25%). The Kappa coefficient was found highest in 1990 (0.95), followed by 2020 (0.94), 2010 (0.91) and 2000 (0.90). The high overall accuracy of more than 96% shows acceptable agreement between the classified image and the ground truth data for the present study.

**Table 3:** Accuracy assessment for classified images.

Classes	1990			2000			2010			2020		
	Producer’s accuracy (%)	User’s accuracy (%)	Kappa coefficient	Producer’s accuracy (%)	User’s accuracy (%)	Kappa coefficient	Producer’s accuracy (%)	User’s accuracy (%)	Kappa coefficient	Producer’s accuracy (%)	User’s accuracy (%)	Kappa coefficient
Forest	98.02	97.06	0.96	95.00	95.48	0.93	97.50	93.75	0.93	96.50	97.97	0.96
Non-forest	96.97	97.96	0.96	95.50	95.02	0.93	93.50	97.40	0.94	98.00	96.55	0.96
Overall accuracy	97.50			95.25			95.50			97.25		
Overall kappa coefficient	0.95			0.90			0.91			0.94		

### Transition and net forest cover change

**Figure 4:**
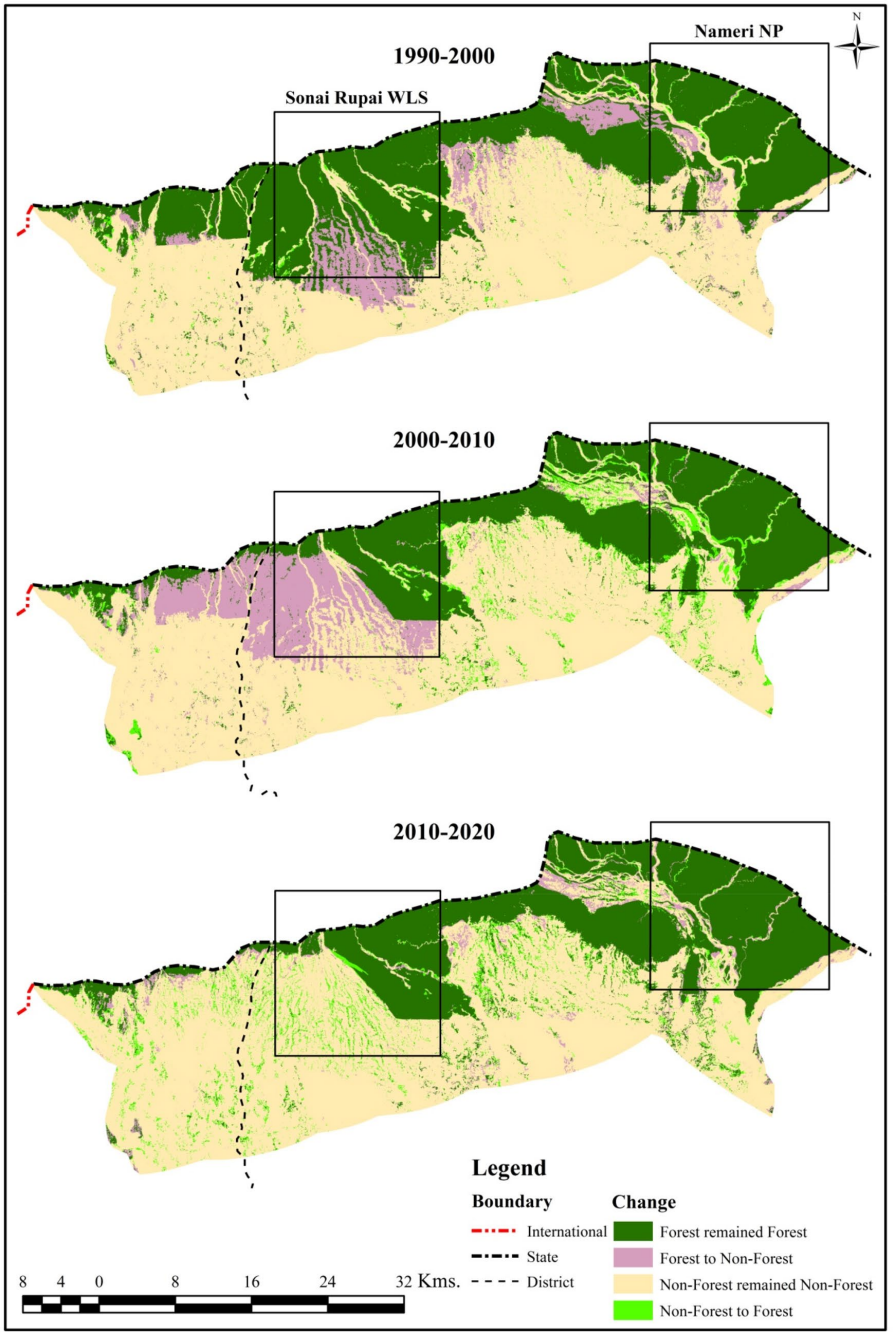
Transition in forest cover (Generated by the authors using ArcGIS 10.3, http://esri.com).

The transition in forest cover from 1990 to 2020 has been classified into four categories viz. Forest remained Forest (FrF), Forest to Non-Forest (FtNF), Non-Forest to Forest (NFtF), and Non-Forest remained Non-Forest (NFrNF). The result shows an area of 594.24 km^2^ (37.25%) as FrF and 149.18 km^2^ (9.35%) as FtNF from 1990 to 2000. Further, an area of 821.62 km^2^ (51.51%) was classified as NFrNF and 30.10 km^2^ (1.89%) as NFtF during the same decade. The FrF declined about 10% and became 433.94 km^2^ (27.20%) from 2000 to 2010, while the FtNF was 190.40 km^2^ (11.94%). Besides, the NFrNF was 893.78 km^2^ (56.03%), while the conversion of NFtF was 77.02 km^2^ (4.83%). From 2010 to 2020, the FrF slightly increased to 455.10 km^2^ (28.53%) due to an increase in NFtF with 93.16 km^2^ (5.84%). The conversion of FtNF decreased by 3.50%, but the NFrNF consistently increased to 991.02 km^2^ (62.13%). A general trend of declining FrF from 1990 to 2010 and a slight increase from 2010 to 2020 was observed. Further, a considerable decline in FtNF from 11.94% to 3.50% was found between 2000–2010 to 2010–2020. Another significant observation was a perceptible continuous gain in NFtF during the last four decades (Table 4 & Fig. 4).

**Table 4:** Transition in forest cover (1990 to 2020).

Category	1990–2000		2000–2010		2010–2020	
	Area (km^2^)	Area (%)	Area (km^2^)	Area (%)	Area (km^2^)	Area (%)
Forest remained forest	594.24	37.25	433.94	27.20	455.10	28.53
Forest to non-forest	149.18	9.35	190.40	11.94	55.86	3.50
Non-forest to forest	30.10	1.89	77.02	4.83	93.16	5.84
Non-forest remained non-forest	821.62	51.51	893.78	56.03	991.02	62.13
**Total**	**1595.14**	**100**	**1595.14**	**100**	**1595.14**	**100**

**Figure 5:**
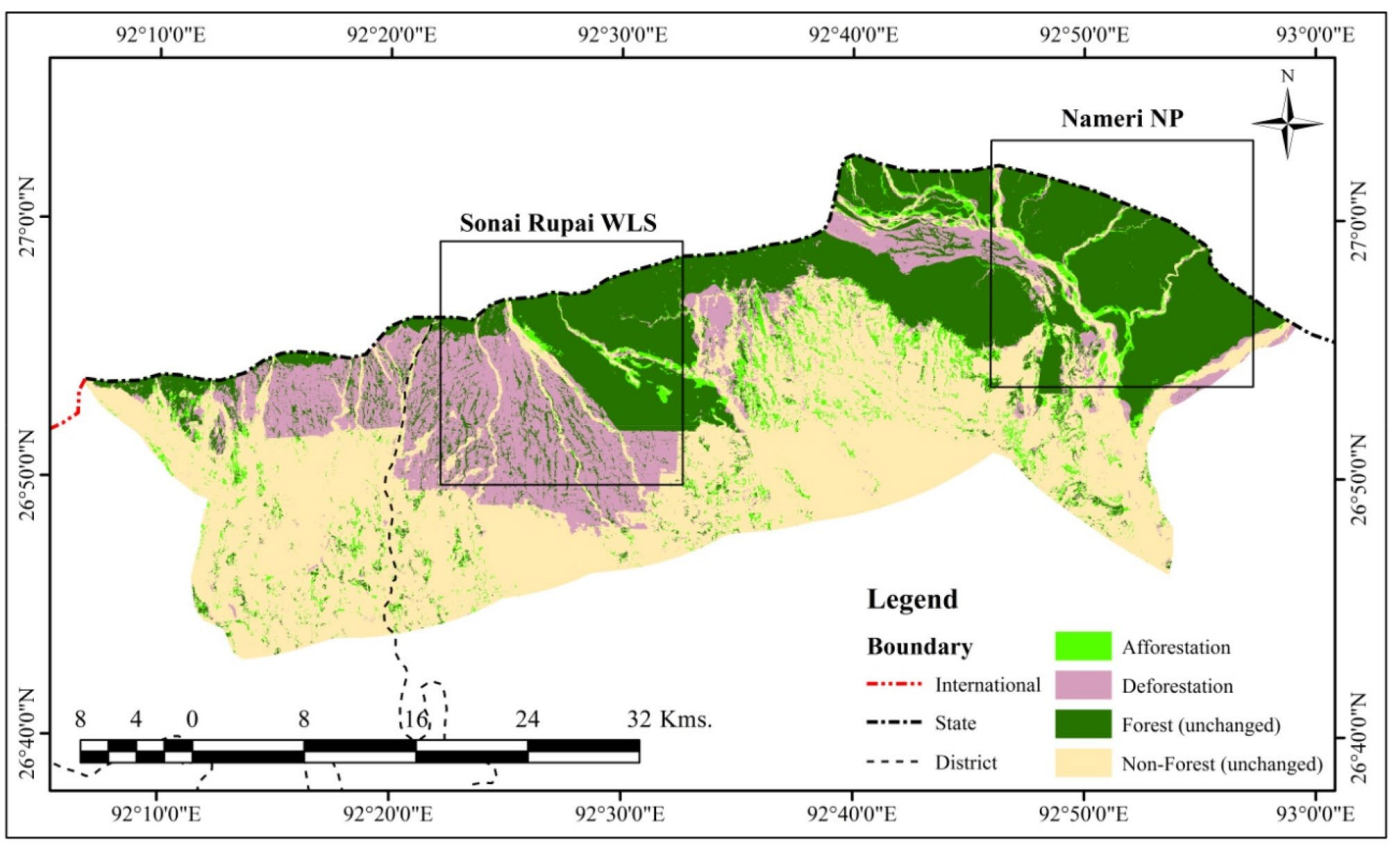
Net change in forest cover map (Generated by the authors using ArcGIS 10.3, http://esri.com).

The final thematic map shows the net change in forest cover of four categories viz. forest (unchanged), non-forest (unchanged), deforestation, and afforestation in the study area from 1990 to 2020. The results show an area of 460.76 km^2^ (28.89%) as forest (unchanged) and 764.21 km^2^ (47.91%) as non-forest (unchanged) during the last forty years. On the other hand, large-scale deforestation of 282.67 km^2^ (17.72%) and afforestation of 87.50 km^2^ (5.48%) was found (Table 5 & Fig. 5). However, the State has registered an increase in forest cover over the past decade that may be attributed to the plantations outside the forest areas, mainly along the roadside. The study also observed a marginal forest cover gain via natural regeneration in the forested areas and along the river valleys.

**Table 5:** Net change in forest cover (1990 to 2020).

Category	Forest cover dynamics (1990–2020)	
	Area (km^2^)	Area (%)
Afforestation	87.50	5.48
Deforestation	282.67	17.72
Forest (unchanged)	460.76	28.89
Non-forest (unchanged)	764.21	47.91
**Total**	**1595.14**	**100.00**

## Discussion

The results reveal a considerable loss of forest cover in the study area, accounting for 7.47% between 1990 to 2000 and 7.11% between 2000 to 2010 with a substantial increase of 2.34% between 2010 to 2020. The overall accuracy of 95.25 – 97.50% and Kappa coefficient of 0.90–0.95 show almost perfect agreement of the forest cover classification matrix^36^. Similar findings of forest cover loss in and around the study area have been reported by Saikia et al*.*^12^ in Nameri NP; Srivastava et al*.*^15^ in Sonitpur District of Assam; Kushwaha & Hazarikha^24^ in Kameng and Sonitpur Elephant Reserves and Balasubramanian et al*.*^37^ in Bura Chapori Wildlife Sanctuary of Nagaon district, Assam. The transition in the forest cover map shows a declining trend of FtNF and a noticeable increase in NFtF between 1990 to 2020. Further, we found large-scale deforestation, accounting for 17.72% in the study area during the past forty years.

Forest loss is driven by factors like commodity production, forestry, agriculture, wildfire, and urbanization^38^. Globally, most of the forest disturbances are associated with commodity-driven deforestation, followed by forestry, shifting agriculture, wildfire, and intensification and expansion of urban centers while in tropical regions, shifting agriculture and commodity-driven deforestation are the major drivers of forest loss^39^. Deforestation is mainly a concern for the developing countries of the tropics^40^ as it is diminishing the areas of the tropical forests^41^. According to Hansen et al*.*^42^ deforestation accounted for 32% of global forest loss in the tropics from 2000 to 2012. In India, the major drivers of forest cover loss are shifting cultivation along with encroachment for agricultural land, mining, quarrying, expansion of settlements, dam construction and illegal logging^43^. In North-East India, the major drivers of forest loss are growing population, agricultural expansion, and dependence on forest resources including fuelwood consumption, logging, and encroachment^44^. In Assam, rapid forest cover changes have occurred in about 33% of the area between 1972 to 1999. Contiguous stretches of forests have been converted to agricultural lands and human settlements, especially in the areas bordering Arunachal Pradesh and Nagaland^45^.

Protected areas are considered to be the principal defense against forest loss^46^. However, the ever-increasing human population in the fringes of protected areas is degrading the forest ecosystems and daily encroachments gradually decrease the buffer zones and the forested areas. A prominent example is the Gir National Park of India, the last bastion of the Asiatic Lion—a meter-gauze railway runs through the park, a state expressway and 3 temples^47^. In this study, we also found significant hotspots of deforestation in Sonai-Rupai WLS and Nameri NP of the Sonitpur district, mainly due to illegal encroachments for human habitation and conversion into cultivable lands. Similar findings have been also reported in the earlier studies^11,12,15,24^. The human settlements around the protected areas have also suffered a significant forest cover losses, resulting in increased incidences of human-wildlife conflicts^24^. Apart from encroachments and expansion of agricultural lands, cutting of trees for timber and fuelwood are the other contributing factors of forest cover loss in the protected areas. Consequently, the wildlife in these areas is facing numerous threats over time^48^. In 2016, the forest officials reported a case of illegal entry and cutting of trees at 24th Mile Camp under Sonai-Rupai WLS^49^. Hence, the probability of more such cases inside the protected areas cannot be rule out. Saikia et al*.*^12^ has also reported large-scale deforestation due to the conversion of forests into agricultural land and firewood extraction activities in Nameri NP from 1973 to 2007. During the recent decade, the State in general and the protected areas, in particular, has recorded an incremental gain in forest cover. As per the FSI^10^, forest cover in the State increased by 221.51 km^2^ in two years, i.e., 2017 to 2019. The forest cover also increased by 14.61 km^2^ and 9.52 km^2^ in Sonitpur and Udalguri district, which forms a significant part of the present study area.

## Conclusion

The assessment of forest cover change is essential to understand forest dynamics like afforestation and deforestation in an area. Forests are home to various rare and threatened taxa of flora and fauna and need suitable strategies for conservation. The conservation of biological diversity is gaining importance globally, and multiple countries and agencies are working towards the maintenance of forest health and thereby conservation and re-introduction of different extinct species in protected areas. The national parks and wildlife sanctuaries are the ultimate areas designated for the conservation of important flora and fauna throughout the world. However, the problem of encroachments, timber, and fuelwood extraction from such sites has increased rapidly during the past decades. Hence, the Ministry of Environment, Forests, and Climate Change (MoEFCC), the Government of India have notified Eco-Sensitive Zones (ESZs) to regulate and manage the activities around protected areas, national parks, and wildlife sanctuaries. The Nameri NP and Sonai-Rupai WLS (under the study area) are known for mega-herbivores such as Asian Elephant, Indian Bison as well as several carnivores, including Leopard. The endangered Great Indian One-Horned Rhino was also sighted in the area until 1983^50^. But, the present findings of about 17% of the area under deforestation from these areas appear to be alarming. Therefore, the Assam Forest Department, Government of Assam, while realizing the increasing activities in protected areas, have submitted a proposal for declaring ESZ around the Sonai-Rupai WLS in 2014. However, the 41st ESZ Expert Committee Meeting for declaration of ESZ held through video conferencing on 23rd to 24th June 2020 recommended for finalization of the draft notification. Further, the committee advised the State Government to submit the Conservation Plan on the riverine corridor within a month^51^. This study concludes with a suggestion for the early declaration of Sonai-Rupai WLS and its adjoining areas as ESZ to control and minimize the present trend of deforestation. Besides, the perceptions of the local community towards the protected areas as their common property^46^ need to be changed through awareness and education.
